# Influence of Myocardial Hemorrhage on Staging of Reperfused Myocardial Infarctions With T_2_ Cardiac Magnetic Resonance Imaging: Insights Into the Dependence on Infarction Type With Ex Vivo Validation

**DOI:** 10.1016/j.jcmg.2018.01.018

**Published:** 2018-04-18

**Authors:** Guan Wang, Hsin-Jung Yang, Avinash Kali, Ivan Cokic, Richard Tang, Guoxi Xie, Qi Yang, Joseph Francis, Songbai Li, Rohan Dharmakumar

**Affiliations:** a Department of Radiology, The First Affiliated Hospital of China Medical University, Shenyang, China; b Biomedical Imaging Research Institute, Cedars-Sinai Medical Center, Los Angeles, California; c Department of Biomedical Engineering, Guangzhou Medical University, Guangzhou, China; d Department of Veterinary Medicine, Louisiana State University, Baton Rouge, Louisiana; e David Geffen School of Medicine, University of California, Los Angeles, California.

**Keywords:** acute myocardial infarction, cardiac magnetic resonance, hemorrhage, inflammation, iron, preclinical study

## Abstract

**OBJECTIVES:**

This study sought to determine whether T_2_ cardiac magnetic resonance (CMR) can stage both hemorrhagic and nonhemorrhagic myocardial infarctions (MIs).

**BACKGROUND:**

CMR-based staging of MI with or without contrast agents relies on the resolution of T_2_ elevations in the chronic phase, but whether this approach can be used to stage both hemorrhagic and nonhemorrhagic MIs is unclear.

**METHODS:**

Hemorrhagic (n = 15) and nonhemorrhagic (n = 9) MIs were created in dogs. Multiparametric noncontrast mapping (T_1_, T_2_, and T_2_*) and late gadolinium enhancement (LGE) were performed at 1.5- and 3.0-T at 5 days (acute) and 8 weeks (chronic) post-MI. CMR relaxation values and LGE intensities of hemorrhagic, peri-hemorrhagic, nonhemorrhagic, and remote territories were measured. Histopathology was performed to elucidate CMR findings.

**RESULTS:**

T_2_ of nonhemorrhagic MIs was significantly elevated in the acute phase relative to remote territories (1.5-T: 39.8 ± 12.8%; 3.0-T: 27.9 ± 16.5%; p < 0.0001 for both) but resolved to remote values by week 8 (1.5-T: −0.0 ± 3.2%; p = 0.678; 3.0-T: −0.5 ± 5.9%; p = 0.601). In hemorrhagic MI, T_2_ of hemorrhage core was significantly elevated in the acute phase (1.5-T: 17.7 ± 10.0%; 3.0-T: 8.6 ± 8.2%; p < 0.0001 for both) but decreased below remote values by week 8 (1.5-T: −8.2 ± 3.9%; 3.0-T: −5.6 ± 6.0%; p < 0.0001 for both). In contrast, T_2_ of the periphery of hemorrhage within the MI zone was significantly elevated in the acute phase relative to remote territories (1.5-T: 35.0 ± 16.1%; 3.0-T: 24.2 ± 10.4%; p < 0.0001 for both) and remained elevated at 8 weeks post-MI (1.5-T: 8.6 ± 5.1%; 3.0-T: 6.0 ± 3.3%; p < 0.0001 for both). The observed elevation of T_2_ in the peri-hemorrhagic zone of MIs and the absence of T_2_ elevation in nonhemorrhagic MIs were consistent with ongoing or absence of histological evidence of inflammation, respectively.

**CONCLUSIONS:**

Hemorrhagic MIs are associated with persisting myocardial inflammation and edema, which can confound staging of hemorrhagic MIs when T_2_ elevations alone are used to discriminate between acute and chronic MI. Moreover, given the poor prognosis in patients with hemorrhagic MI, CMR evidence for myocardial hemorrhage with persistent edema may evolve as a risk marker in patients after acute MI.

staging myocardial infarctions (MIs), particularly differentiating the acute from the chronic phase of MI, is a common requirement for making clinical decisions. Over the past decade, cardiac magnetic resonance (CMR) has evolved to fill this need. Specifically, key studies have demonstrated that an approach combining both late gadolinium enhancement (LGE) and T_2_-weighted CMR can be used to stage MI (1). Although LGE CMR (2) is the gold standard for accurately detecting irreversible myocardial injury (3,4), recent concerns about gadolinium-based contrast agents present challenges to their use in patients with severe renal dysfunction (5). Several studies have shown that native (contrast-free) T_1_ mapping can be used to characterize MIs as well. Notably, the capability of native T_1_ mapping for detecting acute MI based on a significant amount of edema within acute MI territories has been demonstrated (4-11). Recent studies by Kali et al. (12,13) have provided evidence that native T_1_ mapping at 3.0-T, when combined with T_2_-based imaging, can also enable characterization of chronic MIs without contrast agents. Thus, the collective evidence in the literature suggests that T_2_ CMR is critical for imaging-guided staging of MIs when used in conjunction with contrast-enhanced infarct imaging (e.g., LGE) or contrast-free CMR (e.g., native T_1_ mapping at 3.0-T).

A growing body of evidence supports the notion that hemorrhagic MIs are associated with prolonged inflammation, well after the formation of scar tissue (11,14,15). Because inflammation is typically associated with T_2_ elevations, it is unclear whether the resolution of T_2_ signals to baseline levels is sufficient to stage all (hemorrhagic and nonhemorrhagic) MI types. Moreover, given that the outcomes in post-MI patients are increasingly recognized to be dependent on MI type (16-19), there is strong motivation to reassess the capabilities of T_2_ CMR in the staging of both hemorrhagic and nonhemorrhagic MIs.

We hypothesized that staging of MIs as acute (within 1 week of MI) or chronic (8 or more weeks after MI) based on resolution of T_2_ CMR must take into account whether reperfused MIs are hemorrhagic or nonhemorrhagic. We tested our hypothesis in clinically relevant animal models of acute (<1 week after MI) and chronic MIs (8 weeks after MI) at 1.5- and 3.0-T, along with histopathologic analysis.

## MATERIALS AND METHODS

### ANIMAL PREPARATION AND HANDLING.

**FIGURE 1:**
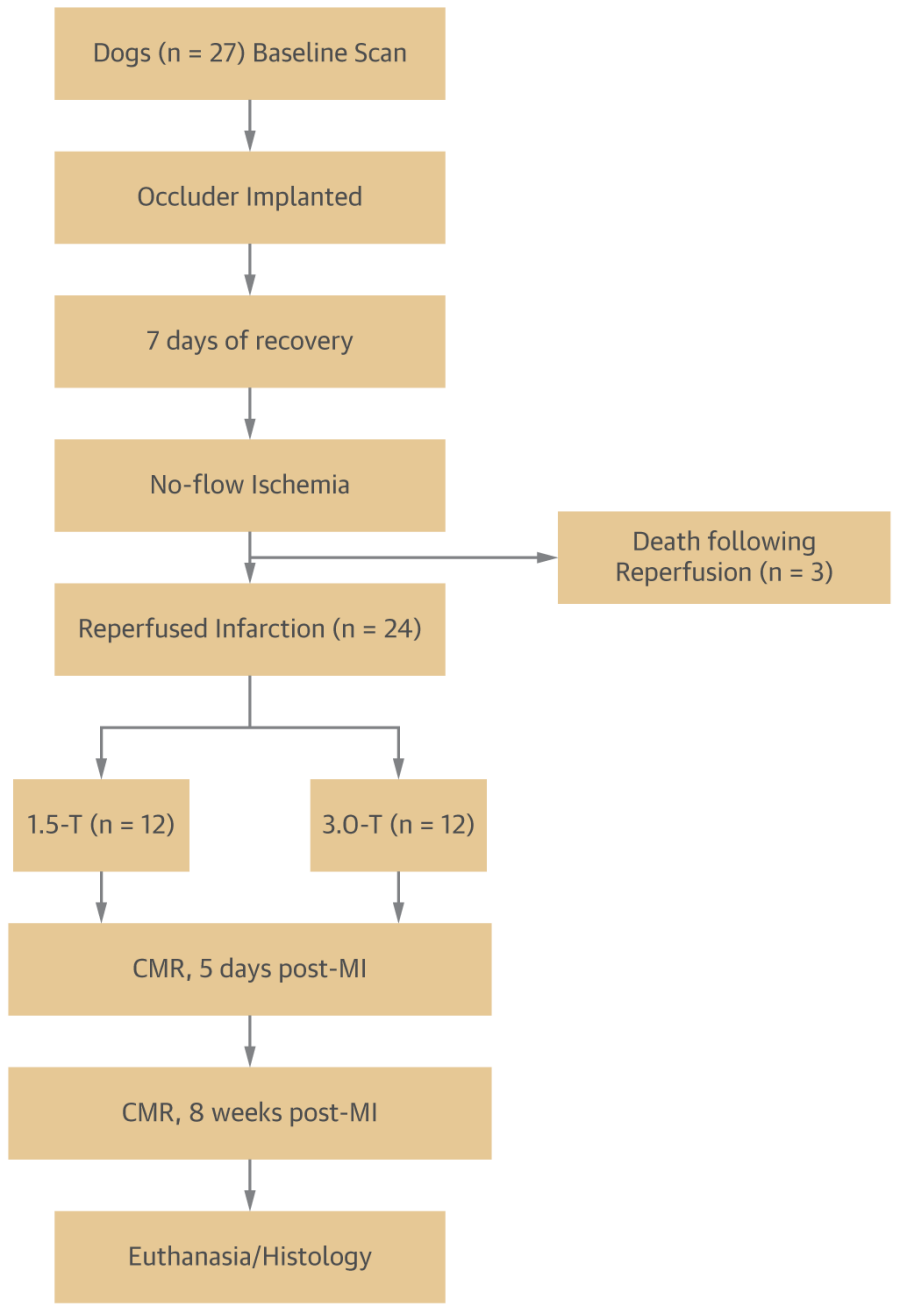
Time Line for Canine Studies After a 3-h no-flow ischemia and then reperfusion, 24 animals were randomized for imaging at 1.5- and 3.0-T, on day 5 and week 8 post-MI. After the week 8 CMR study, animals were sacrificed and their hearts harvested for histology and immunohistochemistry. CMR = cardiac magnetic resonance; MI = myocardial infarction.

Dogs (n = 27; weight 20 to 25 kg) were studied according to protocols approved by the institutional animal care and use committee. A time line of the canine studies is shown in Figure 1. Reperfused MI at the left anterior descending coronary artery territory was created with a no-flow ischemia in the left anterior descending coronary artery below the first diagonal for 3 h, followed by reperfusion. Animals were imaged with CMR under general anesthesia and mechanical ventilation on day 5 and week 8 post-MI, hereinafter referred to as the acute and chronic phases of MI, respectively. Animals that survived the MI were randomized for imaging at 1.5-T (1.5-T group) or 3.0-T (3.0-T group). After the week 8 CMR, animals were sacrificed, and their hearts were harvested for histology and immunohistochemistry.

### CMR ACQUISITIONS AND ANALYSIS.

CMR studies were performed in 1.5- and 3.0-T (Espree and Verio, respectively, Siemens Healthcare, Erlangen, Germany) clinical systems. After whole-heart shimming and scouting, electrocardiograph-gated, breath-held, contiguous, slice-matched short-axis T_1_, T_2_, and T_2_* maps and LGE covering the whole left ventricle were acquired. LGE signal intensities and native T_2_, T_2_*, and T_1_ values of hemorrhagic territories (core [Hemo+] and periphery of hemorrhage [Peri-Hemo+] within MI), nonhemorrhagic territories (Hemo−), and remote (Remote) myocardium were measured using commercial software. Details of the imaging sequences and image analyses, including blinding, and interobserver and intraobserver, are provided in the Supplemental Appendix.

### HISTOLOGY AND IMMUNOHISTOCHEMISTRY.

Infarcted territories identified on CMR were visually matched to the infarcted sections delineated by triphenyltetrazolium chloride (TTC) staining using anterior papillary muscle as a landmark. Representative samples of infarcted and remote myocardium from these sections were selected for histopathologic analysis (hematoxylin and eosin [H&E] for tissue damage, Masson trichome for fibrosis, and Perl stain for iron) and immunohistochemistry (MAC387 for newly recruited macrophages). Within the infarcted regions, the presence of yellow-brown stain and iron in Perl stain was used to identify hemorrhagic regions. If infarcted territories showed evidence of MAC387-positive cells, the MI territories were confirmed for the presence of active inflammation in the chronic phase of MI.

### STATISTICAL ANALYSIS.

On the basis of CMR analysis, results were pooled into 8 groups (Supplemental Tables 1 and 2) based on field strength (1.5-T vs. 3.0-T), age of infarction (acute vs. chronic), and MI territory (Hemo+, Peri-Hemo, Hemo−). The data from each group were labeled on the basis of field strength, MI type, and age of MI. Statistical analysis was performed using IBM SPSS Statistics 23 (IBM Corp., Armonk, New York). The interobserver reliability in measuring LGE, T_1_, T_2_, and T_2_* values was assessed using an intraclass correlation coefficient (ICC). Bonferroni corrections were used to adjust the significance level for multiple comparisons. Additional details on statistical analysis are provided in the Supplemental Appendix.

## RESULTS

Twenty-four animals were available for imaging (12 animals at 1.5-T and 12 at 3.0-T; 3 died during reperfusion). On T_2_* CMR, hemorrhage was evident in 15 animals (7 from the 1.5-T group and 8 from the 3.0-T group) and appeared hypointense both in the acute and chronic phases. The corresponding sections from these animals were positive for iron on histopathology. No hemorrhage was evident in the other animals (n = 9; absence of T_2_* loss and iron deposits within MI territories from corresponding histological sections). Some aspects of the T_1_ and T_2_ data from 11 animals have been previously reported (13). On the basis of LGE, a total of 958 imaging slices (LGE, T_2_*, T_2_, T_1_) were identified as positive for MI, but 62 of these images (T_2_*, T_2_, T_1_) were excluded from analysis because of uninterpretable image quality; thus, the final analysis was performed on 896 imaging slices.

**FIGURE 2:**
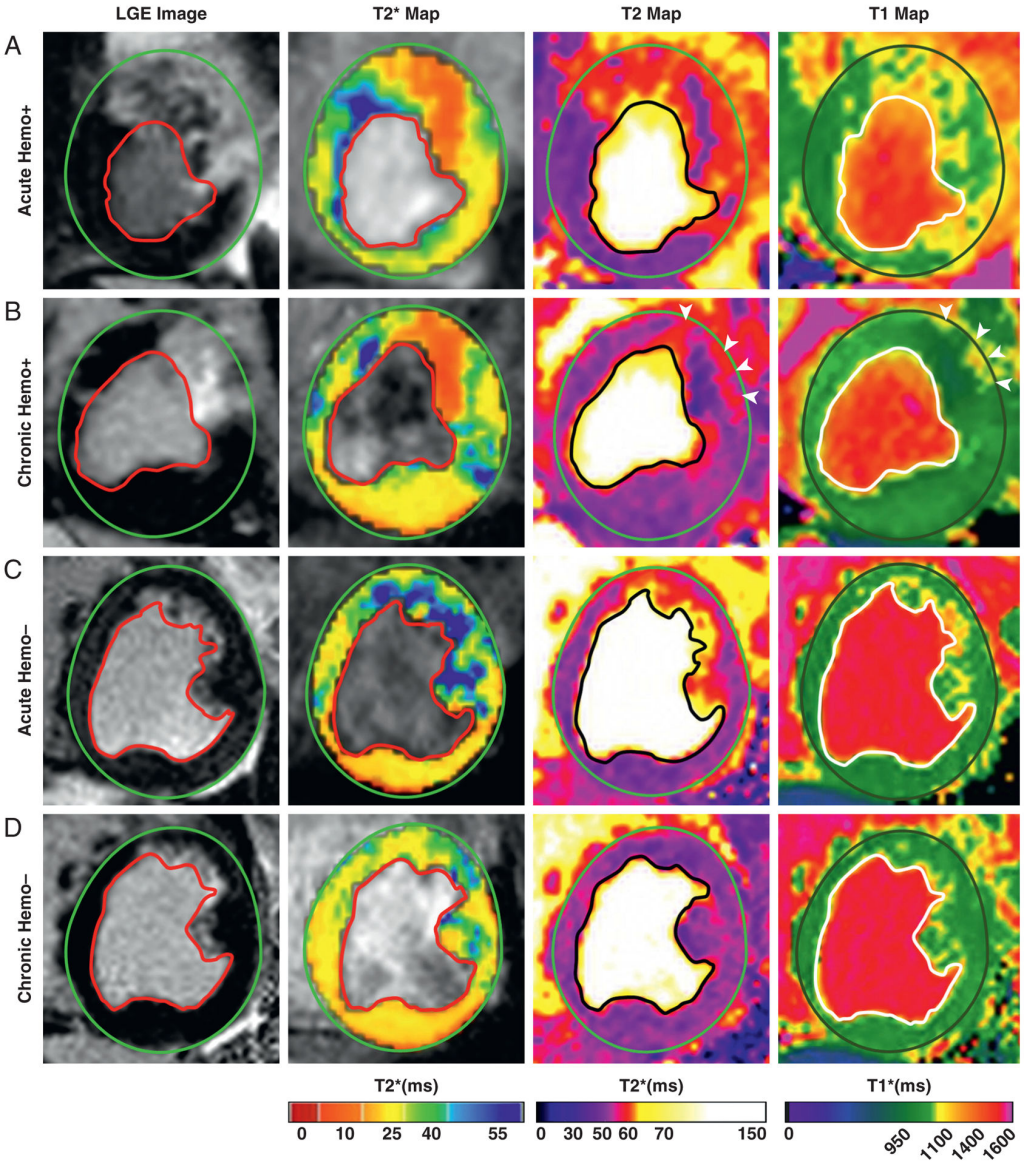
LGE and Noncontrast-Enhanced Relaxation Maps of Canine Hearts in the Acute and Chronic Phases of Reperfused MI at 1.5-T **(A, B)** Representative LGE images and native T_2_*, T_2_, and T_1_ maps acquired 5 days (acute) and 8 weeks (chronic) after reperfusion in a dog with hemorrhagic MI. **(C,D)** Representative LGE images and native T_2_*, T_2_, and T_1_ maps acquired 5 days (acute) and 8 weeks (chronic) after reperfusion in a dog without hemorrhagic MI. LGE = late gadolinium enhancement; MI = myocardial infarction.

**FIGURE 3:**
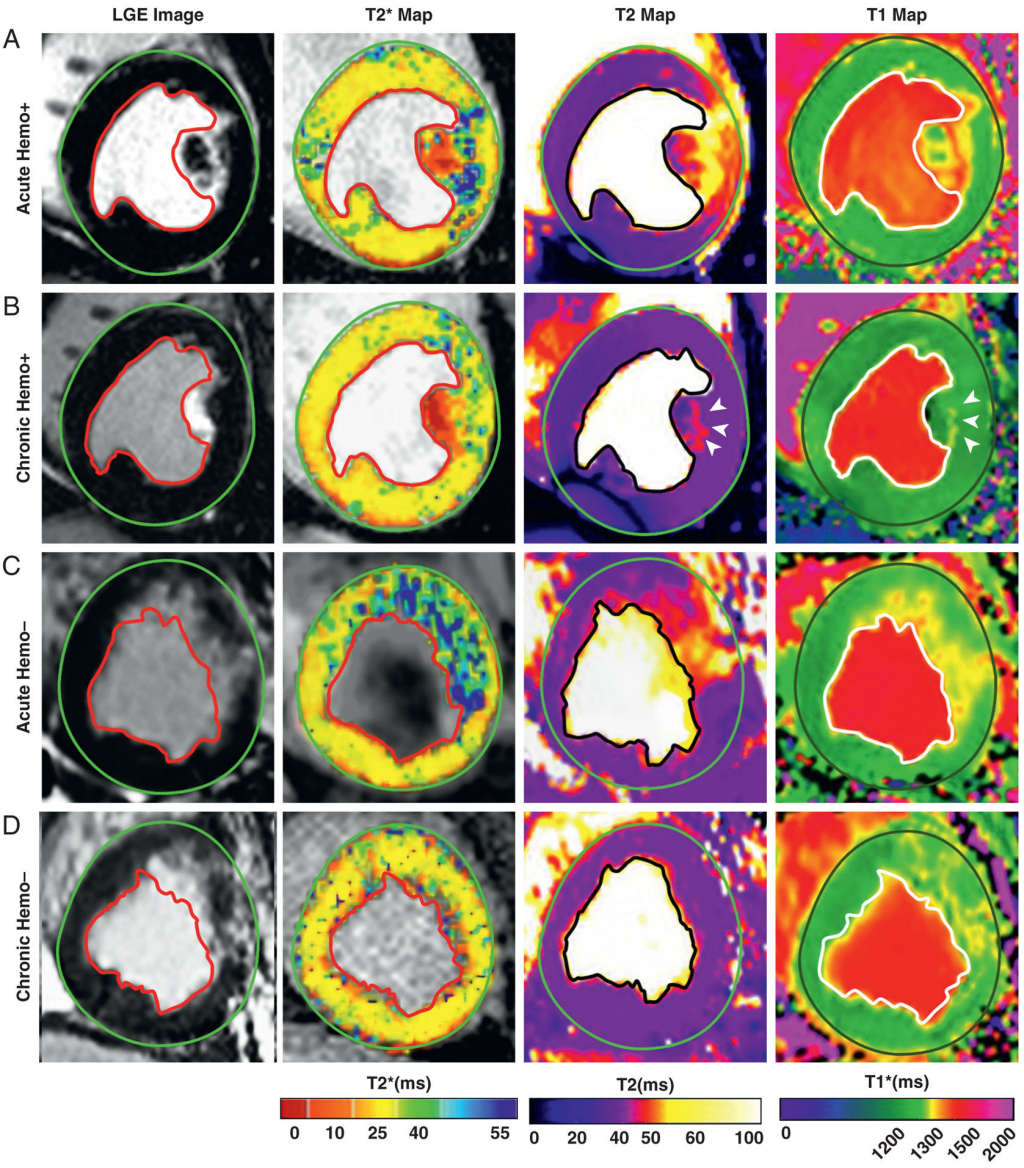
LGE and Noncontrast-Enhanced Relaxation Maps of Canine Hearts in the Acute and Chronic Phases of Reperfused MI at 3.0-T **(A, B)** Representative LGE images and native T_2_*, T_2_, and T_1_ maps acquired 5 days (acute) and 8 weeks (chronic) after reperfusion in a dog with hemorrhagic MI. **(C, D)** Representative LGE images and native T_2_*, T_2_, and T_1_ maps acquired 5 days (acute) and 8 weeks (chronic) after reperfusion in a dog without hemorrhagic MI. LGE = late gadolinium enhancement; MI = myocardial infarction.

Representative LGE images and corresponding noncontrast-enhanced T_2_*, T_2_, and T_1_ maps from animals with and without hemorrhage in the acute and chronic phases of MI, acquired at 1.5- and 3.0-T, are shown in Figures 2 and 3, respectively. Similar to previous studies (14,20), nonhemorrhagic MIs were identified as those MIs without T_2_* loses in the acute phase (Figures 2C and 3C) or chronic phase (Figures 2D and 3D), independent of field strength. These regions were visualized with marked hyperintensities on T_2_ maps at 1.5- and 3.0-T in the acute phase (Figures 2C and 3C, respectively). The same animals followed to the chronic phase did not show any hyperintensities within the MI zone on T_2_ maps at 1.5- or 3.0-T (Figures 2D and 3D, respectively). On T_1_ maps in the acute phase, these regions were visualized with marked hyperintensities at 1.5- and 3.0-T (Figures 2C and 3C, respectively). The infarcted regions of same animals followed to chronic phase of MI were visualized with mild T_1_ hyperintensities at 1.5-T (Figure 2D) and moderate to high T_1_ hyperintensities at 3.0-T (Figure 3D).

Similarly, hemorrhagic MIs were visualized as hypointense regions on T_2_* maps in acute and chronic phases at 1.5-T (Figures 2A and 2B, respectively) and 3.0-T (Figures 3A and 3B, respectively) within the MI territories identified on LGE CMR. On T_2_ maps acquired in the acute phase of MI, hemorrhagic territories identified on T_2_* maps were visualized as hypointense core surrounded by hyperintense rim on T_2_ map at 1.5-T (Figure 2A) and 3.0-T (Figure 3A). Notably, the core of the hemorrhage on T_2_ maps was moderately hyperintense relative to the remote myocardium at both field strengths. In the chronic phase of MI, T_2_ maps showed moderately hyperintense regions surrounding the zone of T_2_* loss at 1.5-T (Figure 2B) and 3.0-T (Figure 3B). However, the core territories of MI (i.e., regions showing T_2_* loss) appeared to be mildly hypointense relative to the remote territories at both field strengths. In T_1_ maps, hemorrhage appeared hypointense relative to perihemorrhagic or nonhemorrhagic zones in the acute phase and hypointense relative to the remote myocardium in the chronic phase at 1.5- and 3.0-T.

The interobserver variability measures (ICCs) for all subset of measurements were >0.8. Mean and 95% confidence interval of ICC scores are provided in Supplemental Table S3. Mean LGE, T_2_*, T_2_, and T_1_ values of hemorrhagic, peri-hemorrhagic, nonhemorrhagic, and remote regions; respective relative differences with respect to the remote myocardium (ΔLGE [%], ΔT_2_ [%], ΔT_1_ [%], ΔT_2_* [%]); and absolute differences for T_1_, T_2_, and T_2_* (ΔT_2_, ΔT_1_, ΔT_2_*), along with comparisons, are given in Supplemental Tables 1 (1.5-T) and 2 (3.0-T).

## LGE AND NATIVE MULTIPARAMETRIC MAPPING OF HEMORRHAGIC AND NONHEMORRHAGIC MI AT 1.5- AND 3.0-T

### LGE.

The effect of the age and composition of the MI as well as field strength on LGE, and the relative change in LGE (%) are shown in Supplemental Figure 1. Details are summarized here.

### Core hemorrhagic territories.

The relative changes of LGE signal intensities of hemorrhagic territories in the acute phase was significantly lower than those in the chronic phase at 1.5- and 3.0-T (p < 0.005). Consistent with previous reports (14), the acute MI territories with hemorrhage also showed evidence of microvascular obstruction on LGE, which was absent in the chronic MI territories with a history of reperfusion hemorrhage.

### Peri-hemorrhagic territories.

There were no significant differences in the relative changes of LGE signal intensities between the acute and chronic phases in the peri-hemorrhagic territories at 1.5- and 3.0-T (p = 0.105 at 1.5-T; p = 0.061 at 3.0-T), although there was a trend toward higher relative signal intensities in the chronic phase than in the acute phase at both field strengths.

### Nonhemorrhagic territories.

There were no significant differences in the relative changes in LGE signal intensities between the acute and chronic phases in the nonhemorrhagic territories (p = 0.414 at 1.5-T; p = 0.775 at 3.0-T).

## NATIVE T_2_

**FIGURE 4:**
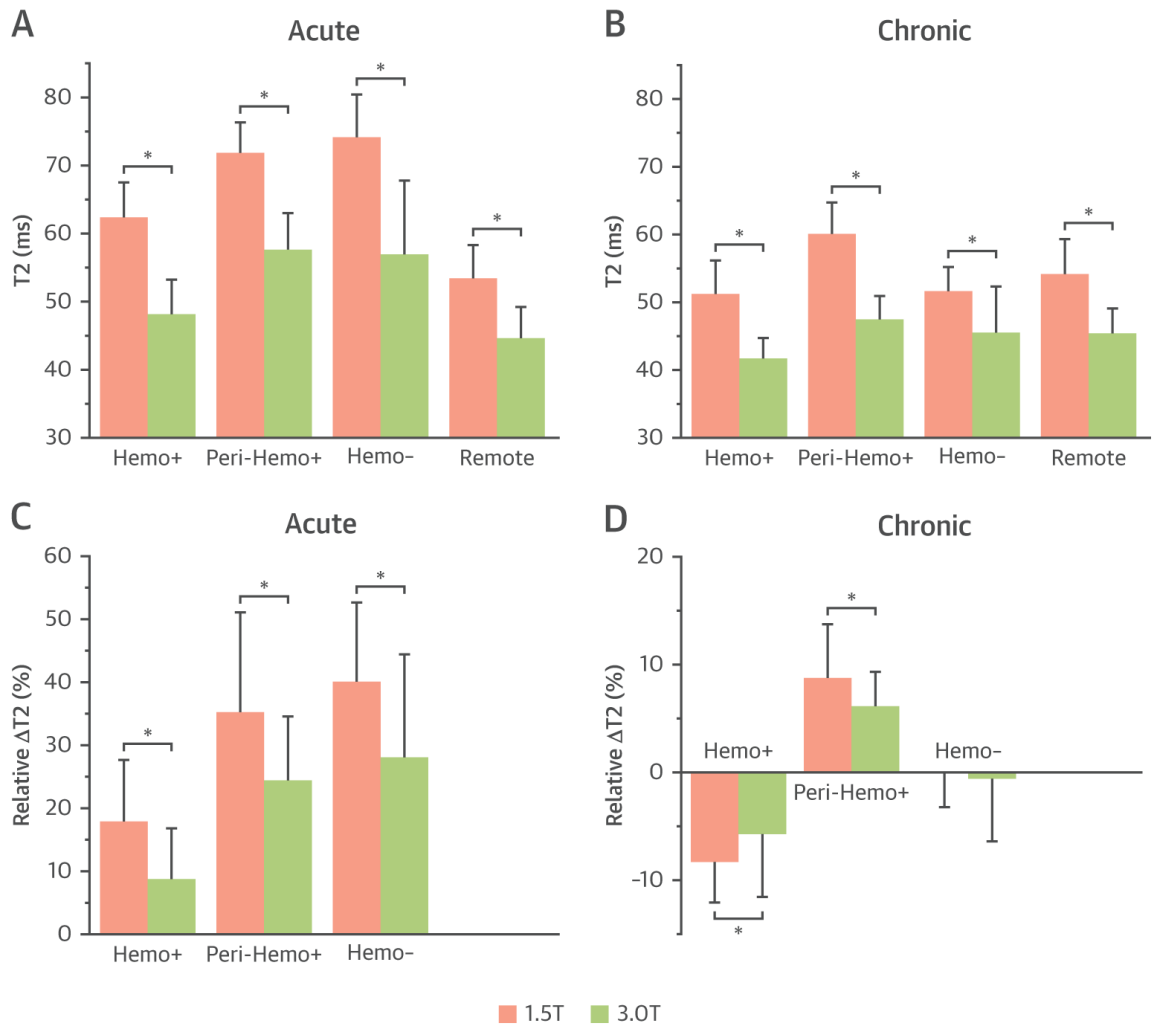
Noncontrast-Enhanced T_2_ of Infarct and Remote Territories and Corresponding Δ T_2_ (%) in the Acute and Chronic Phases of MI at 1.5- and 3.0-T At both field strengths, T_2_ and ΔT_2_(%) of all MI territories (Hemo+, Peri-Hemo+, and Hemo−) were significantly higher in the acute phase than in the chronic phase. In the chronic phase, at both 1.5- and 3.0-T, ΔT_2_(%) was negative in the Hemo+ territory, positive in the Peri-Hemo+ territory, and not different from zero in the Hemo− territory. ΔT_2_(%) of Hemo+ and of Peri-Hemo+ MI territories were different between 1.5- and 3.0-T but not Hemo− territory. The magnitude of ΔT_2_(%) of Hemo+ and Peri-Hemo+ territories was field dependent but not of Hemo− territories. Hemo+ = hemorrhagic MI territories; Peri-Hemo+ = peri-hemorrhagic MI territories; Hemo− = nonhemorrhagic MI territories. *p < 0.05. MI = myocardial infarction.

The effect of age and composition of the MI, as well as field strength, on T_2_ and relative ΔT_2_(%) are shown in Figure 4. T_2_ of all MI and remote territories were significantly higher at 1.5-T compared to 3.0-T (p < 0.005). At 1.5- and 3.0-T, T_2_ of all MI territories were higher in the acute phase than in the chronic phase. Other findings are summarized here.

### Core hemorrhagic territories.

Mean T_2_ of acute hemorrhagic MI territories was higher than mean T_2_ of remote myocardium at both field strengths (p < 0.005). However, in the chronic phase, mean T_2_ of hemorrhagic MI territories were lower than mean T_2_ of remote myocardium at both field strengths (p < 0.005). Mean ΔT_2_ of hemorrhagic zone in the acute phase was significantly higher than that in the chronic phase at both field strengths (p < 0.005 for both).

### Peri-hemorrhagic territories.

In the acute and chronic phases of MI, mean T_2_ of peri-hemorrhagic territories was significantly higher than mean T_2_ of remote territories at both field strengths (p < 0.05). Mean T_2_ of peri-hemorrhagic territories in the acute phase was significantly higher than that in the chronic phase (p < 0.005 at 1.5- and 3.0-T). Mean T_2_ of hemorrhagic territories in the acute and chronic phases of MI were significantly lower than mean T_2_ of peri-hemorrhagic territories at both field strengths (p < 0.005).

### Nonhemorrhagic territories.

In the acute phase, mean T_2_ of nonhemorrhagic acute MI territories was significantly higher than mean T_2_ of remote territories at both field strengths (p < 0.005). However, in the chronic phase, there was no difference in mean T_2_ between infarcted and remote myocardium (p = 0.720 at 1.5-T; p = 0.636 at 3.0-T). Notably, mean ΔT_2_ of nonhemorrhagic territories in the acute phase was significantly greater than that in the chronic phase (p < 0.005 at 1.5- and 3.0-T). Moreover, in the acute phase, mean ΔT_2_ of peri-hemorrhagic regions was not significantly different from mean ΔT_2_ of nonhemorrhagic regions (p = 0.32 at 1.5-T; p = 0.10 at 3.0-T). However, in the chronic phase, mean ΔT_2_ of peri-hemorrhagic regions was significantly higher than mean ΔT_2_ of nonhemorrhagic regions (p < 0.005 at 1.5- and 3.0-T).

### NATIVE T_2_* AND T_1_.

The effect of age and composition of MI on relative ΔT_2_*(%) and ΔT_1_(%) are shown in Supplemental Figures 2 and 3, respectively, at 1.5- and 3.0-T. T_2_* of all MI and remote territories were significantly higher at 1.5-T compared to 3.0-T (p < 0.005), whereas T_2_* of all MI and remote territories were significantly lower at 1.5-T compared to 3.0-T (p < 0.005). Other findings are summarized in the following.

### Core hemorrhagic territories.

Mean T_2_* of hemorrhagic territories was significantly lower relative to remote myocardium in the acute and chronic phases of MI at and 3.0-T (p < 0.005). At 1.5-T, mean T_2_* of hemorrhagic territories in the acute phase was larger than in the chronic phase of MI (p < 0.005), but this difference was not observed at 3.0-T (p = 0.239). In the acute phase of MI, mean T_1_ was significantly higher in the hemorrhagic territories compared to the remote myocardium (p < 0.005 at 1.5- and 3.0-T). However, in the chronic phase, mean T_1_ of hemorrhagic MI territories was lower than mean T_1_ of the remote myocardium at 1.5-T (p < 0.005) but not different from one another at 3.0-T (p = 0.443). At and 3.0-T, T_1_ of hemorrhagic MI territories was significantly larger in the acute phase than in the chronic phase (p < 0.0001).

### Peri-hemorrhagic territories.

Mean T_2_* in the acute phase of MI was significantly higher in the perihemorrhagic regions relative to remote territories (p < 0.005 at 1.5- and 3.0-T); however, there was no difference in the chronic phase (p = 0.426 at 1.5-T; p = 0.287 at 3.0-T). T_2_* of hemorrhagic territories was significantly lower than T_2_* of peri-hemorrhagic territories at both phases of MI (p < 0.005 at 1.5- and 3.0-T). T_2_* of peri-hemorrhagic MI territories was significantly larger in the acute phase than in the chronic phase (p < 0.05 at 1.5- and 3.0-T). T_1_ of the peri-hemorrhagic territories in the acute and chronic phases was significantly higher than in the remote myocardium (p < 0.005 at 1.5- and 3.0-T). At 1.5-T, mean T_1_ of peri-hemorrhagic territories was not different between acute and chronic phases of MI (p = 0.111) but was lower in the chronic phase compared to the acute phase at 3.0-T (p = 0.042).

### Nonhemorrhagic territories.

Mean T_2_* of nonhemorrhagic territories was significantly higher relative to remote myocardium in the acute phase of MI at and 3.0-T (p < 0.005 for both) but was not different from remote myocardium in the chronic phases of MI (p = 0.081 at 1.5-T; p = 0.536 at 3.0-T). There was also no significant difference between ΔT_2_* of peri-hemorrhagic and nonhemorrhagic territories when compared for infarct age or field strength (p > 0.20 for all comparisons). At 1.5-T, mean T_2_* of nonhemorrhagic territories in the acute phase was larger than in the chronic phase of MI (p < 0.0001), but this difference was not observed at 3.0-T (p = 0.316). Mean T_1_ of the nonhemorrhagic territories in the acute and chronic phases of MI was significantly higher than mean T_1_ of remote myocardium at 1.5- and 3.0-T (p < 0.005 for all). Notably, mean ΔT_1_ of nonhemorrhagic territories in the acute phase was greater than that in the chronic phase at both field strengths (p < 0.005). There was no significant difference between ΔT_1_ of peri-hemorrhagic and nonhemorrhagic regions at both phases of MI (p > 0.30 at and 3.0-T). T_1_ of nonhemorrhagic MI territories was significantly larger in the acute phase than in the chronic phase (p < 0.006 at 1.5- and 3.0-T).

### HISTOPATHOLOGY OF HEMORRHAGIC AND NONHEMORRHAGIC INFARCTIONS.

**FIGURE 5:**
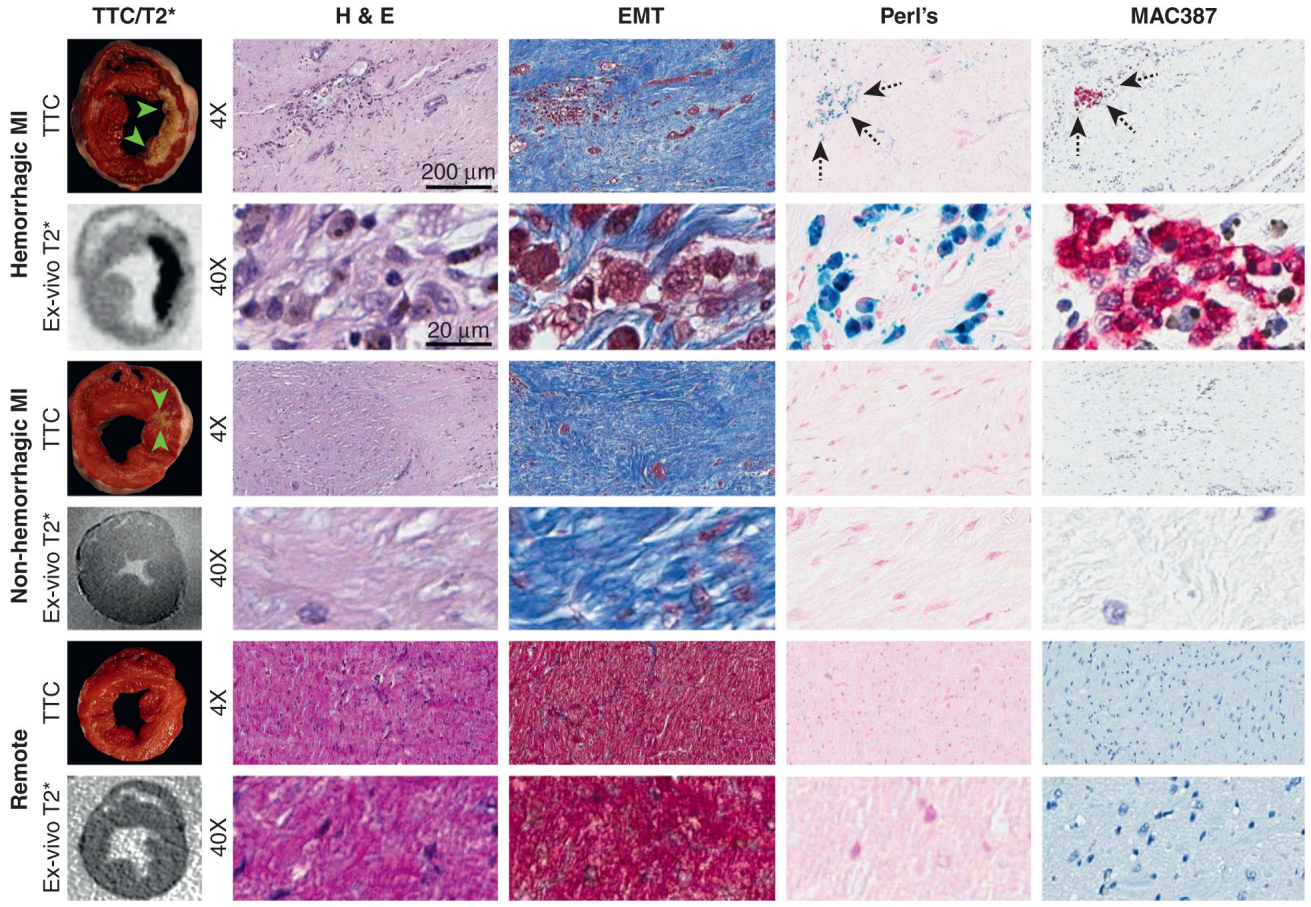
Histopathologic Findings From Myocardial Tissue Section of Hemorrhagic and Nonhemorrhagic Chronic MIs Representative ex vivo images (T_2_*-weighted and corresponding TTC stained sections) from dogs with hemorrhagic and nonhemorrhagic MIs. Remote myocardial sections from a dog with a history of hemorrhagic MI are shown for reference. TTC images were used to localize the infarct zone, and ex vivo T_2_*-weighted images were used to identify whether the infarcted zones were of hemorrhagic origin (based on hypointensities within infarcted myocardium). Histopathology (H&E for tissue damage, EMT for fibrosis, and Perl for iron, at 4× and 40×) and immunohistochemistry (MAC387) analyses were performed from corresponding segments collected from the infarct core and remote sections. H&E = hematoxylin and eosin; EMT = elastin masson trichrome; MI = myocardial infarction; TTC = triphenyltetrazolium chloride.

Representative histopathologic images from animals with hemorrhagic and nonhemorrhagic infarctions sacrificed at week 8 of MI are shown in Figure 5. TTC staining confirmed the presence or absence of infarcted myocardium. Specifically, TTC staining of hemorrhagic territories (identified as described in the following text) showed brownish stain within the infarct core, whereas this was not evident in nonhemorrhagic territories. H&E stains showed evidence of myocardial injury, and elastin masson trichrome stains showed extensive fibrosis within infarcted regions. Perl stains showed substantial localized iron within hemorrhagic territories (identified as MI regions with T_2_* loses on ex vivo images). However, this was not evident in nonhemorrhagic territories (identified as MI regions without evidence of T_2_* loses on ex vivo images) or remote myocardium from hemorrhagic or nonhemorrhagic MIs. Microstructural histopathologic features of remote myocardium obtained in animals with hemorrhagic and nonhemorrhagic MIs did not show evidence of tissue damage on H&E stains and little or no evidence of fibrosis. MAC387 stains showed active infiltration of newly recruited macrophages (<1 week) in regions of iron deposits (from hemorrhagic MIs), but this was not evident in MI regions without iron (nonhemorrhagic MIs).

## DISCUSSION

More than a decade ago, Abdel-Aty et al. (1) demonstrated that T_2_ CMR can be used to discriminate between acute and chronic MIs. Their approach relied on the resolution of T_2_ elevations, associated with edema in the acute phase of MI, to discriminate between acute and chronic MIs. Recent clinical studies reported in the literature, however, have suggested that T_2_ elevations could continue to persist well into the chronic phase of MIs, when edema is expected to be fully resolved. In this study, we examined the capabilities and limitations of T_2_ CMR for discriminating between acute and chronic MIs in 2 important types of reperfused type MIs, namely, those with and those without hemorrhage. Specifically, we tested the hypothesis that the resolution of T_2_ elevations (initially observed in the acute phase) in the chronic phase depends on the infarction type (hemorrhagic vs. nonhemorrhagic MIs) and that staging MIs based on T_2_ CMR requires knowledge of MI type.

Consistent with previous reports on T_2_ between acute and chronic phases of MI, nonhemorrhagic infarct zones showed significantly elevated T_2_ in the acute phase that resolved to remote values by the chronic phase, independent of strength. However, in line with the recent observations by Bulluck et al. (11), this was not observed in the peri-hemorrhagic territories, as these regions showed modest elevation in T_2_ relative to the remote myocardium. This finding was independent of field strength and was suggestive of persistent edema in peri-hemorrhagic territories at week 8 post-MI. Histopathologic analysis confirmed the presence of T_2_ elevations surrounding iron deposits within fibrotic myocardium to be related to ongoing inflammation (recruitment of new macrophages) within the infarct core, providing a rationale as to why T_2_ remains elevated well after the initial hemorrhagic injury. Hemorrhagic territories also showed interesting T_2_ behavior between the acute and chronic phases. In the acute phase, these territories were hypointense relative to the perihemorrhagic regions but hyperintense relative to the remote territories. However, in the chronic phase, they were hypointense relative to the perihemorrhagic regions and remote myocardium, suggesting that at least a partial resolution of edema in the presence of iron deposits enables T_2_ maps to generate negative contrast in chronic hemorrhagic MI territories at 1.5- and 3.0-T. Hence, the findings here support the notion that hypointense chronic MI territories in T_2_ maps at 1.5- and 3.0-T could be interpreted as regions with a history of hemorrhagic damage, especially if they are localized within a rim of T_2_ elevation.

This study also provided additional insight into changes in T_2_* and T_1_ in hemorrhagic and nonhemorrhagic infarctions. First, mean T_2_* of all territories at 3.0-T was lower than at 1.5-T, which is expected because T_2_* relaxation has a strong dependence on field strength (21). We also found that the relative change in T_2_* of all MI territories with respect to remote myocardium was significantly higher in the acute phase than in the chronic phase. This suggests that edema significantly influences T_2_* values, although the hemorrhage imparts an even greater influence than edema on T_2_*. These findings support the possibility that T_2_* relaxation of the infarcted myocardium may be multiexponential, but additional studies are needed to investigate this possibility. Our assessment of the T_1_ of MI territories relative to the remote myocardium also revealed important features. T_1_ of hemorrhagic and peri-hemorrhagic territories was significantly higher at 3.0-T than at 1.5-T. This is consistent with previous reports of field-dependent T_1_ elevations in both acute and chronic phases (9,13). Notably, the T_1_ values in both territories decreased but remained substantially elevated compared to the remote myocardium in the chronic phase of MI, which supports the rationale for using native T_1_ mapping at 3.0-T for identifying chronic MI territories. Previous studies have also demonstrated that, although the T_1_ elevations in the acute phase are likely the result of edema, in the chronic phase they are likely driven by fibrosis (13). However, our findings of T_1_ changes in the hemorrhagic territories provide further insight. Although the pattern of T_1_ decreasing from the acute to the chronic phase was consistent with other territories at 1.5- and 3.0-T, the magnitude of the decrease was significantly different. Specifically, we found that in the chronic phase of hemorrhagic territories, T_1_ values were lower than in the remote myocardium at 1.5-T but not different at 3.0-T. This is likely due to the competing field-dependent effects of iron, which drives T_1_ lower, and replacement fibrosis, which increases T_1_ contrast. This suggests that compared to 1.5-T, at 3.0-T the T_1_ contribution of fibrosis dominates the T_1_ contribution of iron. We also found that in the acute phase of MI, T_1_ within the hemorrhagic territories at both field strengths was significantly reduced, which is likely related to the competing effects between hemorrhagic remnants (decrease T_1_) and interstitial edema (increase T_1_). Our findings of LGE, T_2_*, T_2_, and T_1_ on the types of MI with respect to MI age and how they contribute to image contrast (hyper-, hypo-, or isointense) relative to remote tissue at 1.5- and 3.0-T are summarized in Supplemental Figure 4.

### STUDY LIMITATIONS.

A potential limitation of our study is that our findings are limited to dogs. Thus, the physiological differences between humans and dogs need to be recognized in interpreting the findings. Nonetheless, canine models of reperfused MI have been successfully used to investigate parallel physiological aspects of MI in humans (2,12,13,16,17,22). Moreover, the findings from our study provide insight into how T_2_-based CMR can be used to assess infarct age; however, in practice, clinical history and other imaging metrics may also be used in conjunction with T_2_ CMR to determine the time course of MI.

## CONCLUSIONS

Hemorrhagic MIs are associated with persisting myocardial inflammation and edema, which can confound staging of hemorrhagic MIs when T_2_ elevations alone are used to discriminate between acute and chronic MI. Moreover, given the poor prognosis in patients with hemorrhagic MI, CMR evidence for myocardial hemorrhage with persistent edema may evolve as a risk marker in patients after acute MI.

## Supplementary Material

1

## PERSPECTIVES

**COMPETENCY IN MEDICAL KNOWLEDGE:** Acute versus chronic stage of nonhemorrhagic MI can be determined on the basis of resolution of edema in CMR. However, edema resolution is not a specific discriminator for staging hemorrhagic MI in CMR because chronic hemorrhagic MI territories are associated with prolonged inflammation, which facilitates edema within the myocardial scar. Hence, accurate staging of infarct age based on CMR requires knowledge of whether the index MI was hemorrhagic, which may be gleaned by incorporating T_2_*-based imaging into CMR examinations.

**TRANSLATIONAL OUTLOOK:** The time course of edema and inflammation in the chronic phase of reperfused MIs, specifically contrasting the potential differences between hemorrhagic and nonhemorrhagic MIs, remains to be investigated in patients. In addition, longitudinal CMR studies extending months to years after reperfusion are needed to determine whether a combined evaluation of T_2_ and T_2_* CMR may be more valuable than T_2_ CMR alone to accurately stage MI. Moreover, given the poor prognosis in patients with hemorrhagic MI, CMR evidence for myocardial hemorrhage with persistent edema may evolve as a risk marker in patients after acute MI.

## ABBREVIATIONS AND ACRONYMS

CMR: cardiac magnetic resonance
H&E: hematoxylin and eosin
ICC: intraclass correlation coefficient
LGE: late gadolinium enhancement
MAC387: macrophage marker antibody
MI: myocardial infarction
TTC: triphenyltetrazolium chloride
