# Supplementary material for: Influence of Myocardial Hemorrhage on Staging of Reperfused Myocardial Infarctions With T2 Cardiac Magnetic Resonance Imaging: Insights Into the Dependence on Infarction Type With Ex Vivo Validation
Source: JACC Cardiovasc Imaging. Author manuscript; Available in PMC 2019 May 10. (PMC6510271; doi:10.1016/j.jcmg.2018.01.018)
Supplement: 1 [file NIHMS1525953-supplement-1.docx]

**SUPPLEMENTAL APPENDIX**

**Animal Preparation & Induction of Reperfused Myocardial Infarction**

Each canine was intramuscularly injected pre-anaesthetic tranquilizer innovar (0.4mg/ml fentanyl, 20mg/ml droperidol) at a dose of 1ml/25-50 kg of body weight. The following anesthesia was performed with an intravenous injection of propofol (5.0-7.5 mg/kg), and an endotracheal-intubation gas anesthesia (2.0%-2.5% isoflurane mixed with 100% oxygen). Animals were artificially ventilated at a rate of 1-2 L/min with the respiration rate being continuously adjusted to maintain partial pressure of CO_2_ in arterial blood (Pa CO_2_) within a stable range (30-35 mmHg).

Animal’s chest was opened on the left aspect, at the fourth intercostal space. Animals were instrumented with indwelling aortic and left atrial catheters for blood pressure monitoring and infusion of drugs/withdrawal of fluids during studies. The left anterior descending (LAD) artery was isolated and a hydraulic occluder was affixed at a point 1.0-1.5 cm distal to the first branch. A 20 MHz Doppler flow probe (Crystal Biotech, Northborough, Mass) was positioned distal to the occluder, as close as possible within anatomical constraint, to confirm occlusion. Vital signs monitoring was performed throughout the surgical procedure. The chest was then closed and animals were allowed to recover for a minimum of seven days prior to the first MR study and infarction. Ventricular arrhythmias during surgery or during ischemic/reperfusion were managed with intravenous injection of 20-40 mg lidocaine.

**CMR Acquisitions**

CMR studies were performed in 1.5 T (Magnetom Espree; Siemens Healthcare, Erlangen, Germany) and 3.0T (Magnetom Verio, Siemens Healthcare, Erlangen, Germany) clinical imaging systems. Following whole heart shimming and scouting, ECG-gated, breath-held, contiguous, slice-matched short-axis T1, T2 and T2* maps and LGE covering the whole left ventricle were acquired. Details of the imaging sequences are provided below:

*CMR at 1.5T*: T1 maps were acquired with MOLLI (8 inversion times [TI] with 2 Look-Locker cycles of 3 + 5 images, minimum TI = 120ms, TI increment = 80ms, flip angle = 35°, and readout bandwidth = 1,002 Hz/pixel); T2 maps were acquired using multiple T2 preparations (T2 preparation times =0, 24, and 55ms) with balanced SSFP readouts (flip angle=70°, readout bandwidth=1002 Hz/pixel); T2*-maps were acquired from multi-gradient recalled acquisitions (6 TEs = 3.4ms –18.4ms with ΔTE = 3ms, flip angle = 12°, readout bandwidth =566 Hz/pixel, TR=21ms). LGE image were acquired with PSIR reconstruction (balanced SSFP readouts with TR/TE=3.75/1.75; inversion time was optimized to null viable myocardium; readout bandwidth=1002 Hz/pixel; Flip angle=40^o^). LGE imaging was acquired at 10 minutes after intravenous injection of 0.2mmol/kg gadopentetate dimeglumine (Magnevist, Bayer Healthcare, US). Voxel size for all acquisitions was fixed to 1.5×1.5×8mm^3^.

*CMR at 3.0T*: T1 maps were acquired with MOLLI as at 1.5T, except for readout bandwidth =1371 Hz/pixel. T2 maps were acquired using multiple T2 preparations as at 1.5T except for readout bandwidth of 1371 Hz/pixel. T2*-maps were acquired from multi-gradient recalled acquisitions as at 1.5T with the following differences TR = 20 ms, 6 TEs = 3.3 –13.3 ms (with ΔTE = 2 ms), flip angle = 10°, and readout bandwidth of 1371 Hz/pixel. LGE-PSIR was acquired as in 1.5T except for the following differences: TR/TE = 3.42/1.47 ms; readout band width = 586 Hz/pixel; flip angle = 20^o^). LGE images were acquired 10 minutes after intravenous injection of 0.2mmol/kg gadopentetate dimeglumine. Voxel size for all acquisitions were 1.5×1.5×8mm^3^.

**CMR Image Analysis**

T2* maps were constructed by fitting the multiple gradient-recalled echo data to a mono-exponential decay model. Motion-corrected inline T2 and T1 maps were generated from T2-prepared steady-state free precession images and MOLLI images, respectively. Two expert reviewers (GW and AK), each having more than 5 years of experience in CMR image analysis, blinded to the details of the study analyzed the images in consensus using CVI^42^ (v5.3, Calgary, Canada). Remote myocardium was identified as the region that showed no enhancement on LGE images, and a reference region-of-interest (ROI_Ref_) was drawn respectively in the remote myocardium on LGE, T2* map, T2 map and T1 map images. Infarcted myocardium was defined as the region with mean signal intensity (SI) that is at least 5 standard deviations greater than that of ROI_Ref_ on LGE images,^1^ and the corresponding region-of-interest was denoted as ROI_MI_. Hemorrhagic myocardium was identified as hypointense cores within infarcted territories on the T2* maps using the mean-2SD criterion.^2,3^ Region of interest in the hemorrhagic myocardium (ROI_hemo_) were drawn around the hypointense cores on T2* maps and copied onto the corresponding T2 and T1 maps and LGE images. Peri-hemorrhagic zones (i.e. infarcted regions adjacent to hemorrhage) was defined on LGE as follows: ROI_p-hemo_= ROI_MI_ – ROI_hemo_. This was also copied onto the corresponding T2 and T1 maps. Non-hemorrhagic infarctions were identified as those MI regions without T2* loss in T2* maps. ROIs for non-hemorrhagic infarction were defined to be equivalent to ROI_MI_ (5SD≥ reference ROI) and copied onto the corresponding T2*, T2 and T1 maps for further analysis. Mean signal intensities of LGE, as well as T2*, T2 and T1 values were measured in the infarcted myocardium (including hemorrhagic, peri-hemorrhage, non-hemorrhagic infarcted myocardium) and in remote myocardium. The absolute differences in T2*, T2 and T1 were computed as the respective differences in the values between MI regions of interest and remote myocardium and were labeled as ΔT2*, ΔT2 and ΔT1. The corresponding relative differences were computed by normalizing the absolute difference by value of remote regions and multiplying by 100% and labeled as Relative ΔLGE SI (%), Relative ΔT2*(%), Relative ΔT2(%) and Relative ΔT1(%).

**Statistical Analyses**

Based on CMR analysis, results were pooled into eight groups (Supplemental, Table 1 and Table 2) based on field strength (1.5T vs. 3.0T), age of infarction (acute vs. chronic) and MI territory (Hemo+, Peri-Hemo, Hemo-)). The data from each group was labeled based on field strength, MI type and age of MI. Statistical analysis was performed using SPSS (IBM SPSS Statistics 23). The inter-observer reliability in measuring LGE, T1, T2 and T2* values using CVI^42^ was assessed using intraclass correlation coefficient (ICC). Normality of continuous data was determined by using the Shapiro-Wilk test and quantile-quantile plots. Datasets following normal distributions were compared using analysis of variance (ANOVA) with Student-Newman-Keuls post-hoc analysis. When comparing two groups, comparisons for normal data were also performed using independent-sample t test. Comparison between normal variable and specified test value were performed using one-sample t test. Datasets following a non-normal distribution were compared using the non-parametric Friedman test. When comparing two groups with non-normal data, Mann-Whitney U test was performed. Comparison between non-normal variable and specified test value were performed using one-sample Wilcoxon signed-rank test. Bonferroni corrections were used to adjust the significance level for multiple comparisons.

**References**

1.Amado LC, Gerber BL, Gupta SN, et al. Accurate and objective infarct sizing by contrast-enhanced magnetic resonance imaging in a canine myocardial infarction model. J Am Coll Cardiol 2004;44:2383–9. doi: 10.1016/j.jacc.2004.09.020

2. Bondarenko O, Beek AM, Hofman MB, et al. Standardizing the definition of hyperenhancement in the quantitative assessment of infarct size and myocardial viability using delayed contrast-enhanced CMR. J Cardiovasc Magn Reson 2005;7:481–5. doi: 10.1081/JCMR-200053623 ·

3. Kumar A, Green JD, Sykes JM, et al. Detection and quantification of myocardial reperfusion hemorrhage using T2*-weighted CMR. JACC Cardiovasc Imaging 2011;4:1274-83. doi: 10.1016/j.jcmg.2011.08.016.

| **Supplemental Table 1** T2*, T2, T1 values of hemorrhagic, peri-hemorrhagic and non-hemorrhagic territories and the associated absolute and percent relative differences of the variables with respect to remote myocardium in acute and chronic phases of MI at 1.5T | | | | | | | | |  |
| --- | --- | --- | --- | --- | --- | --- | --- | --- | --- |
|  | | |  | |  | |  |  | |
| **Acute** | | Hemo+ | | Peri-Hemo+ | | Hemo- Remote Myocardium | | |  |
| Relative ΔLGE SI(%) | | 183.6±73.8 | | 418.1±139.4 | | 312.2±127.0 | | |  |
| T2*(ms) | | 22.2±5.5 | | 45.1±6.6 | | 43.6±4.4 39.8±4.0 | | |  |
| ΔT2*(ms) | | -15.3±4.3 (<0, p<0.005) | | 4.2±3.7 (>0, p<0.005) | | 3.4±5.5 (>0, p<0.005) | | |  |
| Relative ΔT2*(%) | | -42.3±11.5 (<0,p<0.0001) | | 10.1±9.3 (>0, p<0.0001) | | 9.6±13.3 (>0, p<0.0001) | | |  |
| T2(ms) | | 62.2±5.3 | | 71.7±4.6 | | 74.0±6.4 53.3±5.0 | | |  |
| ΔT2(ms) | | 9.2±4.9 (>0, p<0.005) | | 18.1±6.7 (>0, p<0.005) | | 19.7±6.0 (>0, p<0.005) | | |  |
| Relative ΔT2 (%) | | 17.7±10.0 (>0, p<0.0001) | | 35.0±16.1 (>0, p<0.0001) | | 39.8±12.8 (>0, p<0.0001) | | |  |
| T1(ms) | | 1066.8±61.2 | | 1148.0±49.7 | | 1166.9±78.2 988±59.8 | | |  |
| ΔT1(ms) | | 73.7±53.7 (>0, p<0.005) | | 158.8±39.1 (>0, p<0.005) | | 169.5±59.4 (>0, p<0.005) | | |  |
| Relative ΔT1 (%) | | 7.1±4.7 (>0, p<0.0001) | | 16.4±4.6 (>0, p<0.0001) | | 17.0±6.1 (>0, p<0.0001) | | |  |
| **Chronic** |  | | |  | |  | | |  |
| Relative ΔLGE SI(%) | | 505.8±100.9 | | 505.3±85.1 | | 348.4±98.8 | | |  |
| T2*（ms） | | 19.7±3.8 | | 37.6±5.9 | | 37.8±5.6 38.4±4.1 | | |  |
| ΔT2*(ms) | | -16.8±4.7 (<0, p<0.005) | | -0.3±1.1 (=0, p=0.426) -0.6±1.3 (= 0, p=0.081) | | | | |  |
| Relative ΔT2*(%) | | -46.8±10.1(<0,p<0.0001) | | -0.7±2.7 (=0, P=0.428) -1.8±3.7 (=0, P=0.071) | | | | |  |
| T2（ms） | | 51.0±5.1 | | 59.9±4.8 | | 51.5±3.7 54.0±5.3 | | |  |
| ΔT2(ms) | | -4.5±2.4 (<0, p<0.005) | | 4.6±2.6 (>0, p<0.005) | | -0.2±2.0 (=0, p=0.720) | | |  |
| Relative ΔT2(%) | | -8.2±3.9 (<0,p<0.0001) | | 8.6±5.1 (>0, P<0.0001) | | -0.0±3.2 (=0, P=0.678) | | |  |
| T1（ms） | | 915.5±109.3 | | 1112.1±61.8 | | 1073.4±103 1044.5±72.1 | | |  |
| ΔT1(ms) | | -95.0±82.0 (<0, p<0.005) | | 83.6±65.1 (>0, P<0.005) | | 88.9±66.6 (>0, p<0.005) | | |  |
| Relative ΔT1(%) | | -9.8±8.6 (<0,p<0.0001) | | 8.2±6.5 (>0,p<0.0001) | | 9.3±5.4 (>0,p<0.0001) | | |  |
| Data are reported as mean ± standard deviation. Acute phase is defined as 5 days after reperfusion and chronic phase is defined as 8 weeks post reperfusion. Hemo+: region of MI containing hemorrhage; Peri-Hemo+: is the area hemorrhagic MI devoid of hemorrhage (peri-hemorrhage area); and Hemo-: non-hemorrhagic area. ΔT2*, ΔT2 and ΔT1 and Relative ΔLGE SI, ΔT2*, ΔT2 and ΔT1 are as defined in text.  Note. <0 and > 0 refer to the direction of the statistical testing for the given variable and = 0 denotes when no difference is observed. | | | | | | | | |  |
|  |  |  |  |  |  |  |  |  |  |

| **Supplemental Table 2** T2*, T2, T1 values of hemorrhagic, peri-hemorrhagic and non-hemorrhagic territories and the associated absolute and percent relative differences of the variables with respect to remote myocardium in acute and chronic phases of MI at 3.0T | | | | | | | | | |  |
| --- | --- | --- | --- | --- | --- | --- | --- | --- | --- | --- |
|  | | |  | | |  | |  |  | |
| **Acute** | Hemo+ | | | Peri-Hemo+ | | | Hemo- Remote Myocardium | | |  |
| Relative ΔLGE SI(%) | | 518.4±246.0 | | 968.6±318.8 | | | 799.0±404.2 | | |  |
| T2*(ms) | | 16.9±4.3 | | | 31.7±5.3 | | 30.7±6.0 28.0±4.1 | | |  |
| ΔT2*(ms) | | -11.7±4.8 (<0, p<0.005) | | | 2.7±2.0 (>0, p<0.005) | | 2.2±5.0 (>0, p<0.005) | | |  |
| Relative ΔT2*(%) | | -39.5±12.3 (<0,p<0.0001) | | | 9.9±7.2 (>0,P<0.0001) | | 9.3±17.4 (>0,P<0.0001) | | |  |
| T2(ms) | | 48.0±5.2 | | | 57.5±5.5 | | 56.8±11.0 44.5±4.7 | | |  |
| ΔT2(ms) | | 3.9±3.5 (>0, p<0.005) | | | 11.0±4.4 (>0, p<0.005) | | 13.7±8.6 (>0, p<0.005) | | |  |
| Relative ΔT2 (%) | | 8.6±8.2 (>0,p<0.0001) | | | 24.2±10.4 (>0,P<0.0001) | | 27.9±16.5 (>0,P<0.0001) | | |  |
| T1(ms) | | 1457.1±167 | | | 1534.9±102.7 | | 1541.9±139.8 1224.0±73.0 | | |  |
| ΔT1(ms) | | 227.2±137.2 (>0, p<0.005) | | | 317.9±80.4 (>0, p<0.005) | | 305.3±96.7 (>0, p<0.005) | | |  |
| Relative ΔT1 (%) | | 20.0±8.8 (>0,p<0.0001) | | | 26.2±6.5 (>0, P<0.0001) | | 27.0±6.2 (>0,P<0.0001) | | |  |
| **Chronic** |  | | | |  | |  | | |  |
| Relative ΔLGE SI(%) | | 1257.6±326.0 | | 1193.2±343.9 | | | 775.1±346.5 | | |  |
| T2*（ms） | | 17.5±4.5 | | | 28.3±3.7 | | 30.2±4.4 30.4±3.6 | | |  |
| ΔT2*(ms) | | -13.1±4.3 (<0, p<0.005) | | | -0.5±2.2 (=0, p=0.287) -0.4±4.0 (=0,P=0.536) | | | | |  |
| Relative ΔT2*(%) | | -43.4±12.6 (<0,p<0.0001) | | | -1.4±8.3 (=0, P=0.368) -1.2±8.9 (=0, P=0.424) | | | | |  |
| T2（ms） | | 41.5±3.2 | | | 47.3±3.6 | | 45.4±6.9 45.3±3.8 | | |  |
| ΔT2(ms) | | -3.1±2.2 (<0, p<0.005) | | | 2.7±1.5 (>0,P<0.005) | | -0.3±3.1 (=0,P=0.636) | | |  |
| Relative ΔT2(%) | | -5.6±6.0 (<0,p<0.0001) | | | 6.0±3.3 (>0,P<0.0001) | | -0.5±5.9 (=0,P=0.601) | | |  |
| T1（ms） | | 1246.0±94.1 | | | 1486.0±77.4 | | 1470.0±105.4 1249.7±64.4 | | |  |
| ΔT1(ms) | | -9.2±78 (=0,P=0.443) | | | 217.1±71.6 (>0,P<0.005) | | 234.1±80.7 (>0,P<0.005) | | |  |
| Relative ΔT1(%) | | -0.6±6.6 (=0,P=0.634) | | | 17.2±6.0 (>0,P<0.0001) | | 19.4±5.5 (>0,P<0.0001) | | |  |
| Data are reported as mean ± standard deviation. Acute phase is defined as 5 days after reperfusion and chronic phase is defined as 8 weeks post reperfusion. Hemo+: region of MI containing hemorrhage; Peri-Hemo+: is the area hemorrhagic MI devoid of hemorrhage (peri-hemorrhage area); and Hemo-: non-hemorrhagic area. ΔT2*, ΔT2 and ΔT1 and Relative ΔLGE SI, ΔT2*, ΔT2 and ΔT1 are as defined in text.  Note. <0 and > 0 refer to the direction of the statistical testing for the given variable and = 0 denotes when no difference is observed. | | | | | | | | | |  |

**Supplemental Table 3** Inter-observer reliability of T2*, T2 and T1 measurements by two-independent readers reported as intraclass correlation coefficients with 95% confidence interval

| Region of Interest | ICC for LGE SI | ICC for T2* | ICC for T2 | | ICC for T1 |
| --- | --- | --- | --- | --- | --- |
| 1.5T/Acute/Hemo+ | 0.915(0.638-0.982) | 0.880（0.782-0.934） | 0.863（0.742-0.927） | 0.826（0.668-0.909） | |
| 1.5T/Acute/Peri-hemo | 0.856(0.443-0.970) | - | 0.812（0.651-0.884） | 0.850（0.711-0.921） | |
| 1.5T/Acute/Hemo- | 0.944(0.748-0.989) | 0.888（0.767-0.946） | 0.856（0.701-0.931） | 0.858（0.705-0.931） | |
| 1.5T/Chronic/Hemo+ | 0.953(0.621-0.981) | 0.845（0.634-0.934） | 0.932（0.839-0.971） | 0.882（0.721-0.950） | |
| 1.5T/Chronic/Peri-hemo | 0.857(0.527-0.962) | - | 0.838（0.724-0.904） | 0.849（0.697-0.921） | |
| 1.5T/Chronic/non-hemo | 0.942(0.766-0.986) | 0.929（0.653-0.954） | 0.844（0.552-0.945） | 0.970（0.915-0.990） | |
| 3.0T/Acute/Hemo+ | 0.905(0.600-0.980) | 0.842（0.728-0.908） | 0.809（0.641-0.898） | 0.890（0.774-0.946） | |
| 3.0T/Acute/Peri-hemo | 0.877(0.508-0.974) | - | 0.887（0.777-0.921） | 0.804（0.674-0.896） | |
| 3.0T/Acute/Hemo- | 0.941(0.763-0.985) | 0.954（0.851-0.949） | 0.906（0.812-0.953） | 0.913（0.837-0.954） | |
| 3.0T/Chronic/Hemo+ | 0.923(0.690-0.981) | 0.974（0.956-0.985） | 0.893（0.804-0.942） | 0.851（0.726-0.918） | |
| 3.0T/Chronic/Peri-hemo | 0.889(0.617-0.971) | - | 0.901（0.764-0.947） | 0.811（0.622-0.908） | |
| 3.0T/Chronic/Hemo- | 0.920(0.712-0.979) | 0.940（0.797-0.938） | 0.903（0.818-0.948） | 0.821（0.667-0.904） | |

**
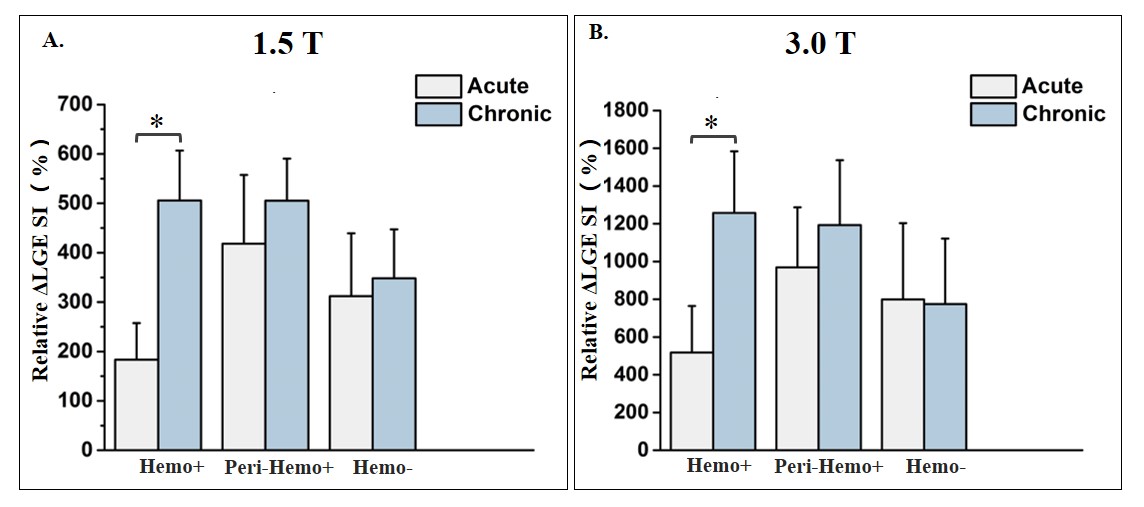
**

**Supplemental Figure 1** *LGE of Infarct and Remote Territories and the Corresponding Δ LGE(%) in the Acute and Chronic Phases of MI at 1.5T and 3.0T.* ΔLGE(%) of hemorrhagic territories were significantly lower in the acute phase than in the chronic phase at 1.5T and 3.0T, but other territories were not different from one another between the difference phases of MI. Hemo+, Peri-Hemo+ and Hemo- denote hemorrhagic, peri-hemorrhagic and non-hemorrhagic MI territories (as defined in text). * denotes p<0.05.

**
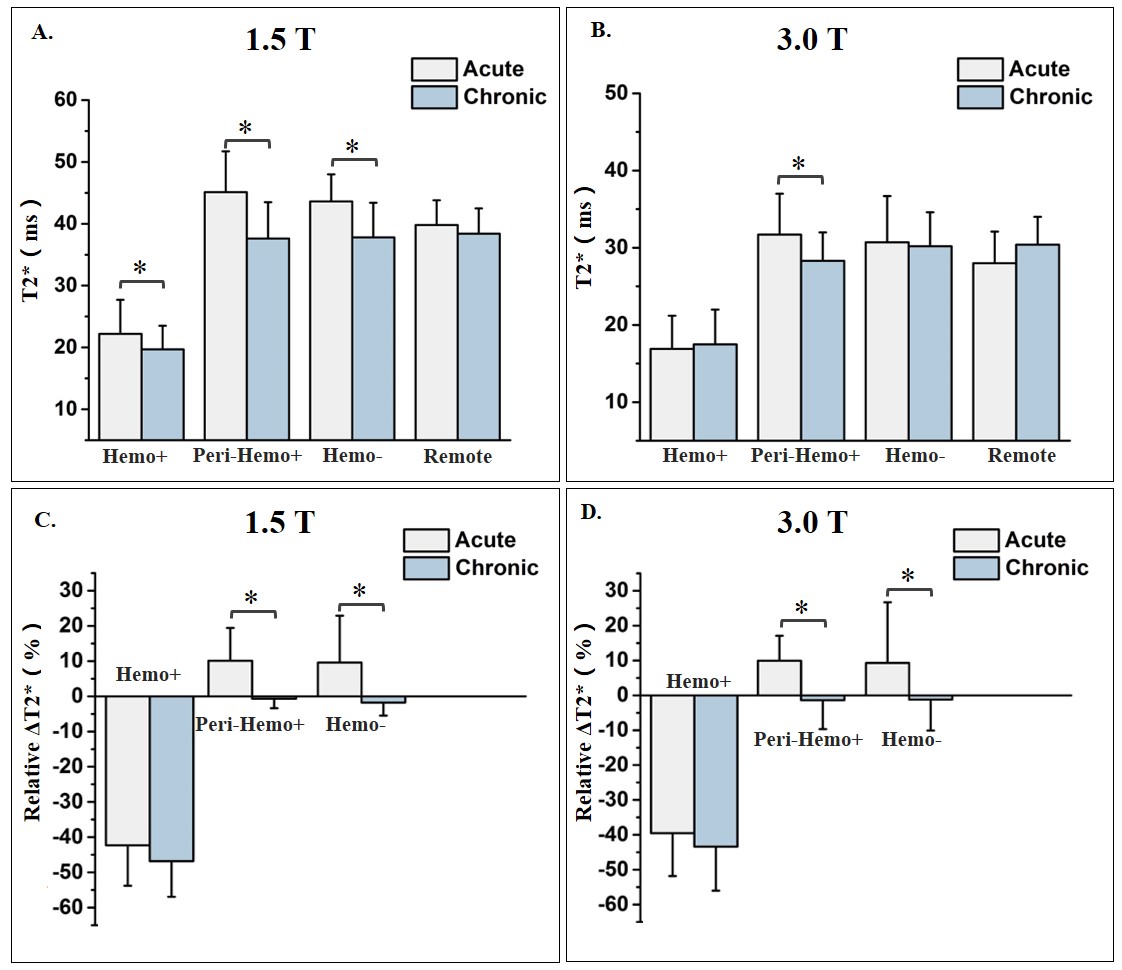
**

**Supplemental Figure 2** *Non-Contrast-Enhanced T2* of Infarct and Remote Territories and the Corresponding ΔT2*(%) in the Acute and Chronic Phases of MI at 1.5T and 3.0T.*  ΔT2*(%) of hemorrhagic territories were negative at 1.5T and 3.0T, other territories were positive in the acute phase but were not different from zero in the chronic phase. Hemo+, Peri-Hemo+ and Hemo- denote hemorrhagic, peri-hemorrhagic and non-hemorrhagic MI territories (as defined in text). * denotes p<0.05.

**
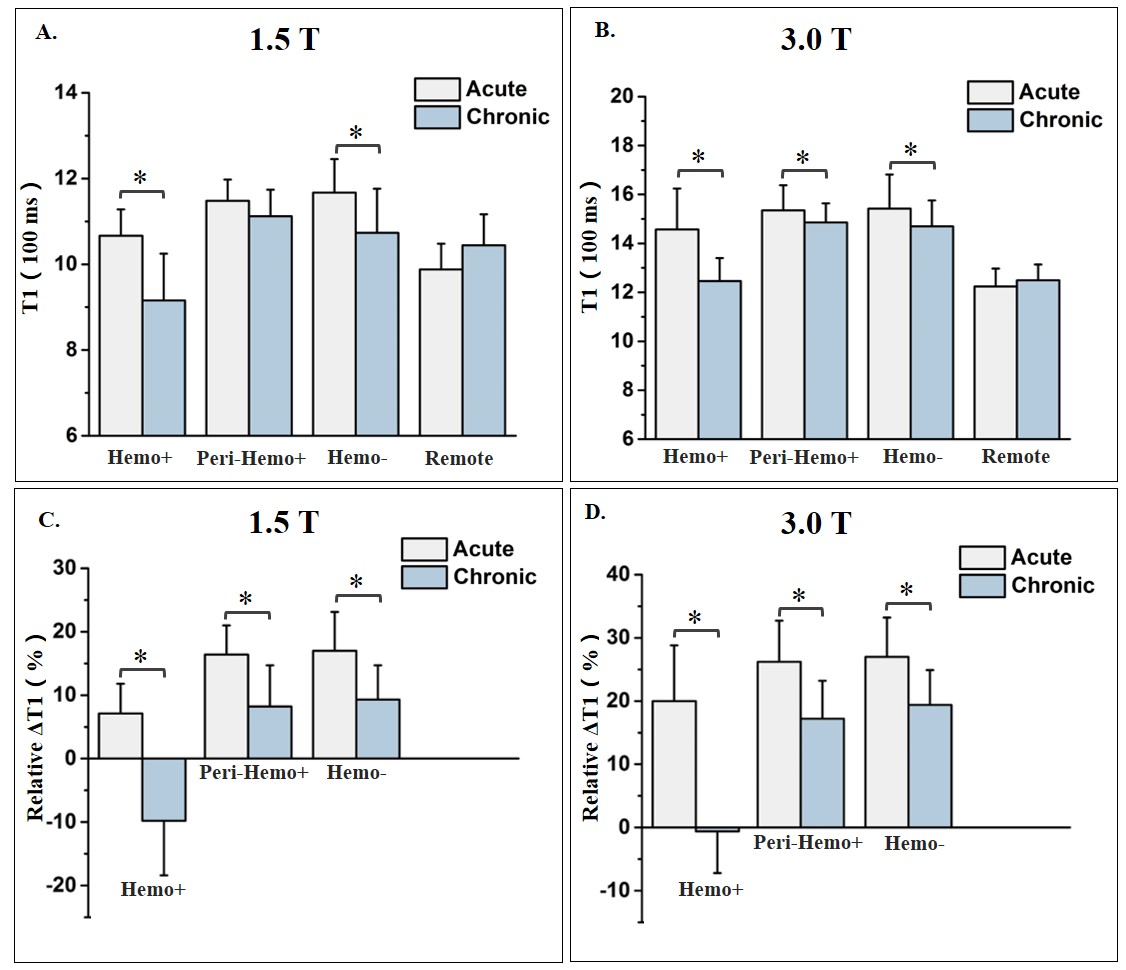
**

**Supplemental Figure 3** *Non-Contrast-Enhanced T1 of Infarct and Remote Territories and the Corresponding ΔT1(%) in the Acute and Chronic Phases of MI at 1.5T and 3.0T.* In the acute phase, ΔT1%) of all MI territories were positive at 1.5T and 3.0T. However, in the chronic phase, ΔT1(%) was negative in the hemorrhagic territory at 1.5T and not different from zero at 3.0T. ΔT1(%) in other territories were positive at both 1.5T and 3.0T. As in Fig. 3, Hemo+, Peri-Hemo+ and Hemo- denote hemorrhagic, peri-hemorrhagic and non-hemorrhagic MI territories (as defined in text). * denotes p<0.05.

**
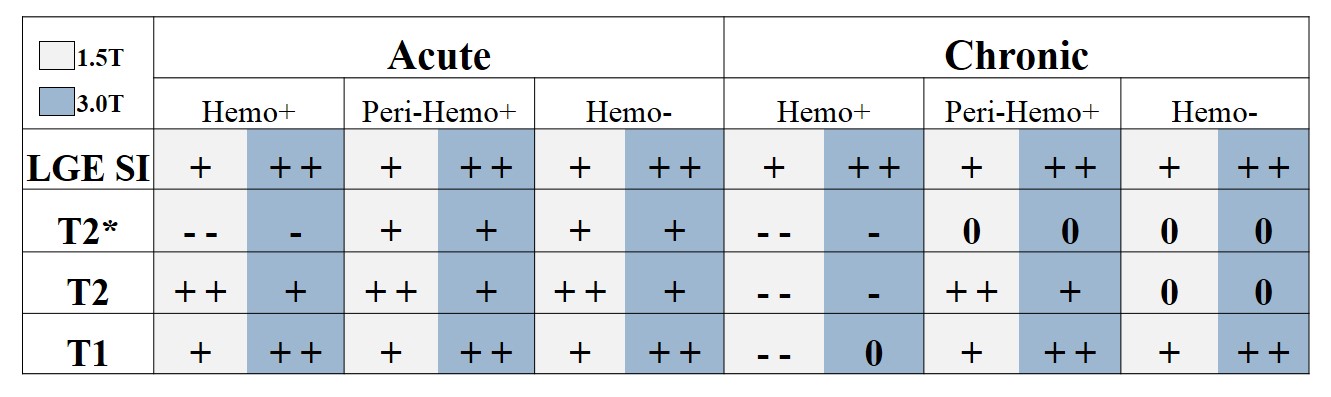
**

**Supplemental Figure 4** *Schematic Summarizing the Dependence of LGE, T2*, T2 and T1 Image Contrast on MI Type at 1.5T and 3.0T.* ‘+’, ‘0’, and ‘-’ denote hyperintense, isointense and hypointense image contrast between the MI territory of interest relative and the remote myocardium. ‘++’ and ‘- -’ denote the magnitude of the hyperintensity or hypointensity, respectively, that are significantly greater at one field strength over the other.
